# Trends and hotspots of publications on ferroptosis: A 10 Year overview

**DOI:** 10.1016/j.heliyon.2023.e18950

**Published:** 2023-08-07

**Authors:** Bingzhou Ji, Guang Yang, Hongfu Jin, Xu Liu, Hengzhen Li, Linyuan Pan, Wenhao Lu, Heyuan Zhu, Yusheng Li

**Affiliations:** a Department of Orthopedics, Xiangya Hospital, Central South University, Changsha, Hunan, China; b Department of Orthopedics, Central Hospital of Loudi, Loudi, Hunan, China

**Keywords:** Bibliometric analysis, Ferroptosis, VOSviewer, CiteSpace, RStudio

## Abstract

**Background:**

Ferroptosis was proposed to be a type of programmed cell death in 2012. Ferroptosis plays a significant role in a variety of illnesses.

**Objective:**

To better understand the direction of future research, we performed a bibliometric analysis to identify research hotspots with a focus on ferroptosis.

**Methods:**

The search terms [TI = “ferroptosis” OR (“GSH” AND “GPX4”) OR “lipid peroxidation” OR “iron homeostasis” OR “iron metabolism”] AND [PY = “2012–2022”] AND [DT = “Article OR Review”] AND [LA = “English”] were used to retrieve publications related to ferroptosis for a bibliometric analysis. We utilized Microsoft Excel to calculate the frequency and proportion of the published articles, VOSviewer to perform a co-occurrence analysis and for visualizing the data, CiteSpace to obtain a timeline of keywords and institutions, and RStudio to calculate citation metrics. As indicated by the analysis, indicators such as the number of publications, the most productive authors and coauthorship status, the distribution of publications by country, favoured journals, the most influential institutions and the most frequently cited documents are reported in this article.

**Results:**

A total of 8009 publications were retrieved from the WOS core collection, and 197 papers published in 2023 were removed from this analysis. The remaining 7812 papers, which included 118 in the WOS collection, were incorporated into the bibliometric study.

**Conclusion:**

The number of annual scientific publications on ferroptosis have been increasing each year. The academic communities represented by Tang, Daolin, Stockwell, Brent R., Wang, Fudi, and Conrad, Marcus were the most authoritative. China, USA, and Germany were the front-runners in the field of ferroptosis. *Free Radical Biology and Medicine* was the largest contributor of ferroptosis-related research, and *Cell* and *Nature* were the most influential journals to publish articles on ferroptosis. Columbia Univ and Univ Pittsburgh were the institutions that received the most attention. Recent research on ferroptosis has been focused on molecular mechanisms, particularly those in the contexts of various diseases, which will be a hotspot of future research. In addition, interdisciplinary ferroptosis and big-data research is expected to be a new frontier.

## 1 Introduction

Ferroptosis was first proposed by Dixon in 2012 and is often accompanied by high iron accumulation and phospholipid peroxidation rates [1]. Ferroptosis can be considered a consequence of cell metabolism. Oxygen and iron are important metabolic drivers that result in the inevitable production of reactive oxygen species (ROS). Cells undergo ferroptosis when phospholipid peroxides (PLOOH), a specific kind of reactive oxygen species (ROS), cannot be effectively removed and thus accumulate, which damages plasma membrane integrity [2]. The substrates for PL peroxidation in cells are PLs with polyunsaturated fatty acyl (PUFA) chains at the sn2 position. PUFA-PLs may be converted to PLOOHs via both enzymatic and nonenzymatic lipid peroxidation mechanisms in the presence of bioactive iron [2]. Through normal cellular metabolism, certain levels of polyunsaturated fatty acid-containing PLs and bioactive iron are produced; therefore, cells leverage monitoring or defence systems to prevent accidental ferroptosis [2]. In human cells, glutathione peroxidase-4 (GPX4) is a unique enzyme capable of catalysing the reduction of phospholipid hydroperoxides into corresponding alcohols [3]. In addition to GPX4-dependent pathways, other mechanisms may inhibit ferroptosis. For instance, enzymes such as FSP1, GCH1, and DHODH are critical for producing metabolites that trap free radicals [[4], [5], [6], [7], [8]]. These metabolites can also abrogate Fenton radical chain reactions and stop the acceleration of lipid peroxidation. Ferroptosis is regulated through several cellular metabolic mechanisms, including lipid metabolism, iron level management, redox homeostasis, and mitochondrial function; factors, such as amino acids; and numerous signalling pathways related to illness. Notably, cancer cells resistant to treatment, especially those of mesenchymal origin and prone to metastasis, are particularly susceptible to ferroptosis. In addition, ferroptosis is the underlying cause of many types of organ damage and a wide variety of degenerative diseases. Therefore, pharmacological regulation of ferroptosis, through both its induction and inhibition, offers considerable promise for the treatment of drug-resistant malignancies, ischaemia-induced organ injuries, and other degenerative disorders connected to excessive lipid peroxidation [9].

Bibliometric analyses can not only help researchers gain a comprehensive overview of a development process and research directions in a particular research area but also be used to identify key authors, journals, institutions and countries in the research field, thereby providing a foundation for future research [10]. The study reports on an assessment of the global trends of ferroptosis research over the past decade and future research directions. To these ends, a scientific information map was generated utilizing CiteSpace, VOSviewer and RStudio. The outcomes of this study will advance fundamental research into ferroptosis and promote the clinical application of ferroptosis.

## 2 Methods

### 2.1 Search strategy and data collection

**Fig. 1:**
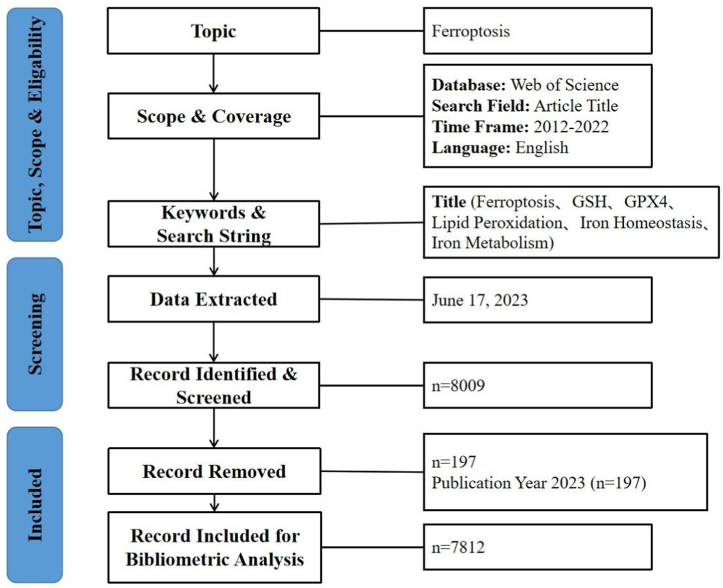
Flow diagram showing the search strategy.

The WOS core database was used to conduct a bibliometric analysis of ferroptosis research published as of June 2023. The search terms [TI = “ferroptosis” OR (“GSH” AND “GPX4”) OR “lipid peroxidation” OR “iron homeostasis” OR “iron metabolism”] AND [PY = “2012–2022”] AND [DT = “Article OR Review”] AND [LA = “English”] (TI: title, PY: publication year, DT: document type, LA: language) were used to search for publications related to ferroptosis. Ferroptosis and subtypes or related signalling pathways were included in the search terms to assure that our findings obtained by completing a publication retrieval task using the search term were thorough and dependable. The title presents a high-level summary of the content of the full text. Our focus on titles was aimed to accurately eliminate articles that fail to meet the purpose of the study. Fig. 1 shows the flowchart of the literature selection process.

### 2.2 Information extraction

We removed publications with errata noted to prevent duplicates when counting the articles and retracted articles, which could have led to articles with false-positive results being included in our research. A bibliometric analysis was carried out on all documents. Four distinct types of software were implemented in this study:

1) Microsoft Excel 2019 to calculate the frequency and proportion of the articles of interest among the published literature;
2) VOSviewer (version 1.6.19) to create and visualize bibliometric networks;
3) CiteSpace (version 5.7. R5) to create timelines for keywords and institutions; and
4) RStudio (version 4.1.2) to calculate citation metrics.

### 2.3 Relevant evaluation metrics

H-index: The H-index is defined as the number (N) of papers published by an author/institution or in a country for which H papers have at least H citations and the other (N–H) papers have fewer than H citations. It can be used to assess the scientific impact of an author/a journal/a country [11].

DF: Dominance Factor, the number of papers for which an author was a first author in coauthored articles [12].

## 3 Results

### 3.1 General information of retrieved publications

**Fig. 2:**
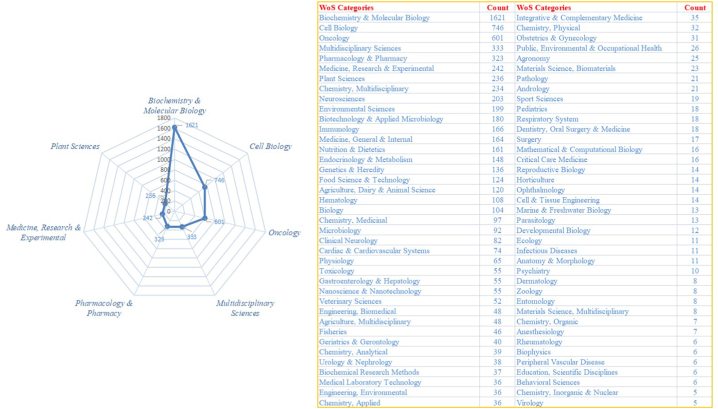
Distribution of WOS categories.

There were a total of 8009 papers retrieved from the WOS database, of which 197 publications were not published in our established time frame. The remaining 7812 publications were included in our analysis. Among these publications, 1119 were review-type papers and 6693 were article-type papers. All articles were published in a journal. By analysing the WOS discipline categories, the articles related to ferroptosis were classified into 118 categories, and the 3 categories with the most ferroptosis articles were biochemistry & molecular biology: 1621, cell biology: 746, and oncology: 601. The WOS disciplinary categories are shown in Fig. 2.

### 3.2 Temporal trends of publications and citations

**Fig. 3:**
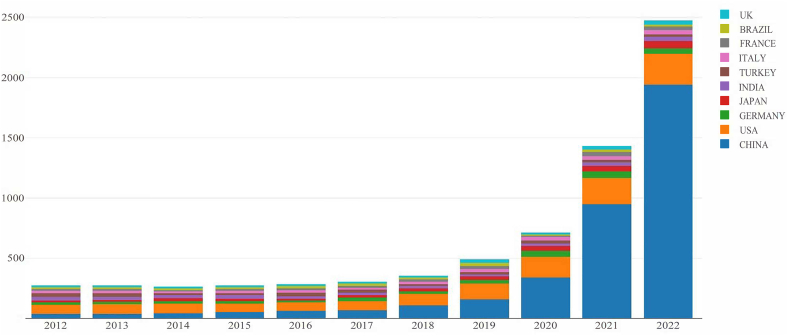
Evolution of country publication contributions to the ferroptosis literature.

Temporal trends of annual publications and citations on ferroptosis research are shown in Table 1. Since the first article was published in 2012, the annual scientific publications on ferroptosis have shown exponential growth. In particular, the number of articles published in 2022 had increased almost twofold compared to the number published in 2021. Hence, it is safe to say that studies related to ferroptosis are receiving increasing attention and show great potential to exert an influence in the future. In 2022, 14363 citations were identified, which the lowest citation period; in contrast, the most citations in any year were found for 2019 (34674 citations) and 2020 (35707 citations). In terms of the average number of citations (AC), the AC was highest for documents published in 2012 and lowest for the papers published in 2022, which was due to the short period between when the article was published and the data were collected. Fig. 3 depicts the annual scientific production rate based on the contributions from different countries. As we can discern from the figure, the USA occupied a leading position in the early stages; however, since 2017, China has overtaken the USA as the country with the highest number of publications.

**Table 1:** Annual scientific productions and citations.

Year	Articles	Citations	Average Citations
2012	353	22467	63.65
2013	384	15911	41.43
2014	349	19512	55.91
2015	365	14711	40.30
2016	350	21586	61.67
2017	366	25865	70.67
2018	426	20151	47.30
2019	550	34674	63.04
2020	782	35707	45.66
2021	1440	34378	23.87
2022	2447	14363	5.87

### 3.3 Productive authors and coauthorship

A total of 31110 authors worldwide have studied ferroptosis, and 103 of these authors published papers as the sole authors. A total of 120 articles (1.54%) were single-author publications, while the others (7692; 98.46%) were multiauthor collaborative publications. Table 2 shows the 15 most prolific authors ranked by number of publications and citations.

**Table 2:** Top 15 authors distributed by publications and citations.

Rank	Author	Publications	Country	Institution	First Author	DF	DF Rank	Author	Country	Institution	H-index	Publications	Total Citations	PY_start
1	Tang, Daolin	61	USA	UT Southwestern Med Ctr	1	0.016	11	Stockwell, Brent R.	USA	Columbia Univ	37	41	24932	2012
2	Kang, Rui	56	USA	UT Southwestern Med Ctr	2	0.036	10	Conrad, Marcus	Germany	Helmholtz Zentrum Munchen	33	42	15329	2014
3	Conrad, Marcus	42	Germany	Helmholtz Zentrum Munchen	3	0.071	6	Dixon, Scott J.	USA	Stanford Univ	19	26	15064	2012
4	Stockwell, Brent R.	41	USA	Columbia Univ	4	0.098	5	Tang, Daolin	USA	UT Southwestern Med Ctr	37	61	13427	2016
5	Wang, Fudi	28	China	Zhejiang Univ	0	0	12	Angeli, Jose Pedro Friedmann	Germany	Helmholtz Zentrum Munchen	13	13	10554	2014
6	Dixon, Scott J.	26	USA	Stanford Univ	4	0.154	2	Kang, Rui	USA	UT Southwestern Med Ctr	36	56	10305	2016
7	Ganz, Tomas	26	USA	Univ Calif Los Angeles	8	0.308	1	Yang, Wan Seok	USA	St Johns Univ	5	5	9143	2012
8	Kagan, Valerian E.	22	USA	Univ Pittsburgh	1	0.045	9	Kagan, Valerian E.	USA	Univ Pittsburgh	19	22	9117	2014
9	Kroemer, Guido	21	France	Univ Paris	0	0	13	Jiang, Xuejun	USA	Mem Sloan Kettering Canc Ctr	18	18	8753	2015
10	Nemeth, Elizabeta	18	USA	Univ Calif Los Angeles	1	0.056	7	Gu, Wei	USA	Columbia Univ	11	14	5330	2015
11	Jiang, Xuejun	18	USA	Mem Sloan Kettering Canc Ctr	1	0.056	8	Kroemer, Guido	France	Univ Paris	18	21	4476	2017
12	Zarkovic, Neven	17	Croatia	Rudjer Boskovic Inst	2	0.118	4	Ganz, Tomas	USA	Univ Calif Los Angeles	21	26	3758	2012
13	Min, Junxia	16	China	Zhejiang Univ	0	0	14	Wang, Fudi	China	Zhejiang Univ	20	28	2917	2014
14	Weiss, Guenter	16	Austria	Med Univ Innsbruck	0	0	15	Gan, Boyi	USA	Univ Texas MD Anderson Canc Ctr	15	15	2761	2018
15	Muckenthaler, Martina U.	16	Germany	Heidelberg Univ	2	0.125	3	Nemeth, Elizabeta	USA	Univ Calif Los Angeles	14	18	2761	2012

**Fig. 4:**
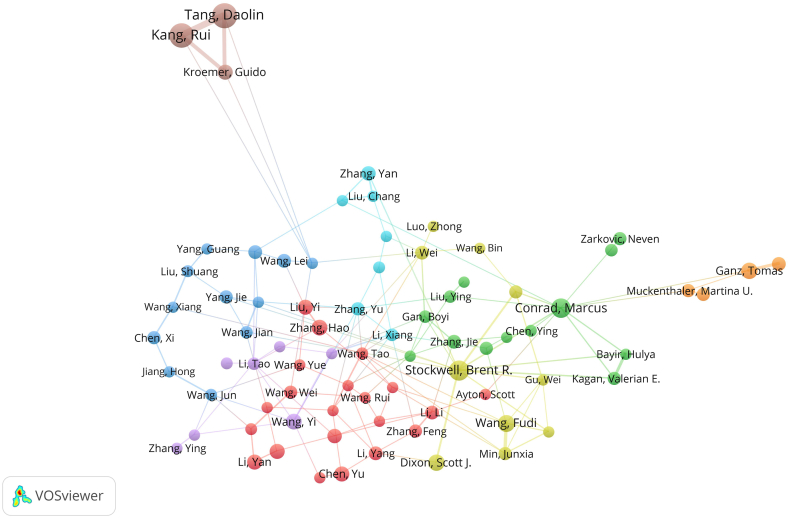
Network and group maps of coauthorship.

The author with the most articles was Tang, Daolin (UT Southwestern Med Ctr, USA), with a total of 61 publications, followed by Kang, Rui (UT Southwestern Med Ctr, USA), with a total of 56 publications, and Conrad, Marcus (Helmholtz Zentrum Munchen, Germany), with a total of 42 publications. The total number of citations for Stockwell, Brent R. (Columbia Univ, USA) exceeded 20000, and was followed by that for Conrad, Marcus (Helmholtz Zentrum Munchen, Germany) at a total of 15329 citations and Dixon, Scott J. (Stanford Univ, USA) with a total of 15064 citations. Based on the rankings of the publications and citations, we concluded that Tang, Daolin; Kang, Rui; Conrad, Marcus, Stockwell, Brent R.; and Wang, Fudi et al. were the most authoritative academics in the field of ferroptosis because these authors were the authors in both the top 15 on the list of publications and citations at the same time. Ganz, Tomas (Univ of California Los Angeles, USA) had the highest DF index. It is worth noting that most of the authoritative authors were from the USA and had relatively low DFs. With the use of VOSviewer software, we identified the authors with more than 10 published articles; these data are presented in Fig. 4. The size of the circles is in proportion to the number of articles published by the authors. The thickness of the connecting lines between authors is proportional to the number of times these authors appeared together in the same publication. The colour of the circles represents collaboration between the authors. Several communication groups were formed by the academics in the field of ferroptosis. As indicated by the data in Table 2, we focused more attention on four academic groups represented by Tang, Daolin; Stockwell, Brent R.; Wang, Fudi; and Conrad, Marcus.

### 3.4 Geographical distribution of publications and citations

**Fig. 5:**
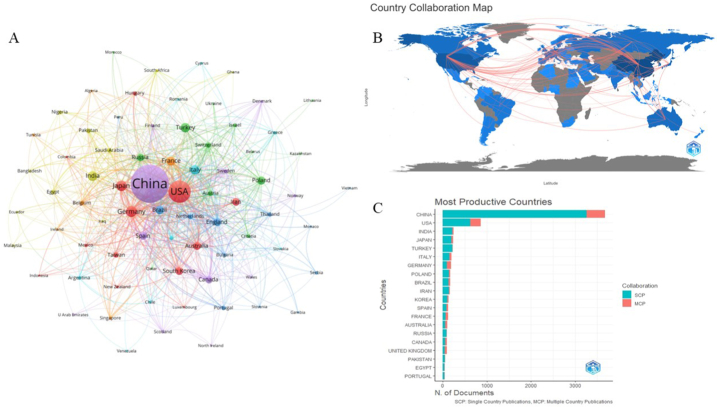
Cooperation among researchers in the field of ferroptosis among countries. (A) Network map showing international collaboration among the authors in different countries. (B) Country collaboration map. (C) Distribution of single-country publications and multiple-country publications.

A total of 116 countries were involved in ferroptosis-related research, with the publications originating largely in China, the USA, India, and Japan. The countries with the highest largest scientific production were China (3674 articles, 85375 citations) and the USA (857 articles, 71345 citations). The top 10 countries ranked on the basis of the number of publications and citations are presented in Table 3. China, as the most prolific country, accounted for 47.03% of the total number of articles published. Germany and the USA were also at the top of the list of the average number of citations, which was much higher than that of other countries, indicating that research from these countries appear to be more popular around the world. Although China is the leader in the number of publications, the average number of citations of papers originating in China lagged behind that of other countries, suggesting that Chinese academics should pay more attention to the quality of published papers. Authors in China, the USA and Germany established a high number of scientific collaborations with authors from other countries, while authors in Turkey and Iran established fewer collaborative relationships with authors from other countries (Fig. 5A and B). The distribution of publications based on coauthors from a single or multiple countries is presented in Fig. 5C.

**Table 3:** Top 10 country distributed by publications and citations.

Rank by publications	Country	Articles	Citations	Average Citations (AC)	Percentage (%)	SCP	MCP	Rank by AC
1	CHINA	3674	85375	23.24	47.03	3263	411	5
2	USA	857	71345	83.25	10.97	626	231	2
3	INDIA	241	4504	18.69	3.08	211	30	6
4	JAPAN	237	6902	29.12	3.03	195	42	4
5	TURKEY	223	2788	12.50	2.85	212	11	10
6	ITALY	194	6471	33.36	2.48	148	46	3
7	GERMANY	189	16689	88.30	2.42	95	94	1
8	POLAND	169	2638	15.61	2.16	140	29	8
9	BRAZIL	167	2671	15.99	2.14	129	38	7
10	IRAN	159	2349	14.77	2.04	139	20	9

### 3.5 Analysis of keywords

Keywords are at the core of an article. We can surmise the research subjects of a certain field and identify the research hotspots and directions by analysing the keywords. The top 10 most frequent author keywords (DE) were ferroptosis, lipid peroxidation, oxidative stress, iron, iron metabolism, hepcidin, inflammation, reactive oxygen species, iron homeostasis, and prognosis. The top 10 most frequent Author Keywords-Plus (ID) were oxidative stress, cell-death, expression, metabolism, mechanisms, iron, death, cancer, activation, and apoptosis. Table 4 shows the specific frequencies of these terms.

**Table 4:** Keywords by frequency.

Rank	Author keywords (DE)	Frequency	Keywords-plus (ID)	Frequency
1	Ferroptosis	2710	Oxidative Stress	1754
2	Lipid Peroxidation	1229	Cell-Death	1186
3	Oxidative Stress	666	Expression	957
4	Iron	603	Metabolism	806
5	Iron Metabolism	346	Mechanisms	679
6	Hepcidin	261	Iron	645
7	Inflammation	213	Death	591
8	Reactive Oxygen Species	197	Cancer	556
9	Iron Homeostasis	195	Activation	549
10	Prognosis	188	Apoptosis	499

**Fig. 6:**
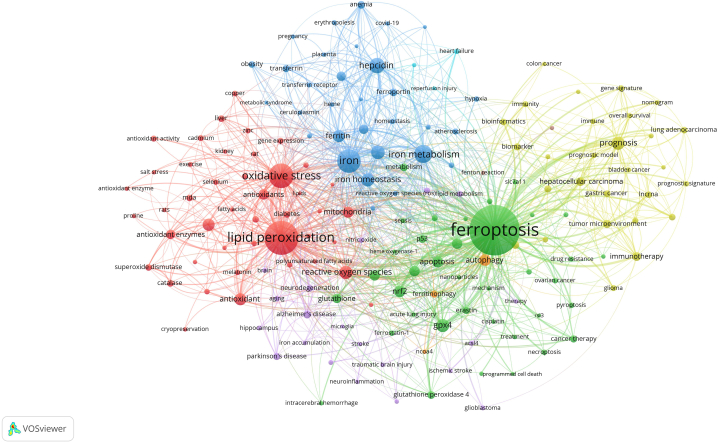
Network map of author keywords.

The network map is based on clusters of co-occurring keywords, which illustrates the fundamental framework of relevant research topics. VOSviewer software was applied to cluster keywords from publications. As a whole, the circles and labels constitute a unit, and various coloured units make up distinct clusters. As shown in Fig. 6, the author keywords that appeared at least 10 times were classified into 4 clusters, which represented 3 different ferroptosis subfields. The keywords associated with the green cluster were related to ferroptosis, GPX4 and glutathione. Notably, GPX4 is thought to be at the centre of ferroptosis regulation [3], and it relies on glutathione to protect cells from ferroptosis by eliminating phospholipid peroxides [13]. The keywords associated with the red cluster were related to lipid peroxidation and oxidative stress. Notably, polyunsaturated fatty acids (PUFAs), especially arachidonoyl and adrenoyl, contain diallyl hydrogen atoms, and these PUFAs are prone to react with ROS, causing lipid peroxidation and leading to ferroptosis [14]. The keywords for the blue cluster were related to iron metabolism. Iron is necessary for ferroptosis, and various iron chelators inhibit ferroptosis. Conversely, an increase in the intracellular pool of unstable iron increases the sensitivity of cells to ferroptosis [15]. Notably, ferroptosis is mediated via two main mechanisms: a Fenton reaction with PUFAs and Fe^2+^ to produce ROS, and Fe^2+^ is a cofactor for certain enzymes that mediate lipid peroxidation [16]. Overall, the keywords for the green, red, and blue clusters represent the three classical metabolic pathways of ferroptosis. The keywords in the yellow cluster were related to on disease treatment and prognostic evaluation, which reflect the role of ferroptosis in clinical treatment. After integrating these results, we concluded that the research directions on ferroptosis can be summarized into two main categories: molecular mechanisms and clinical treatment of various diseases.

**Fig. 7:**
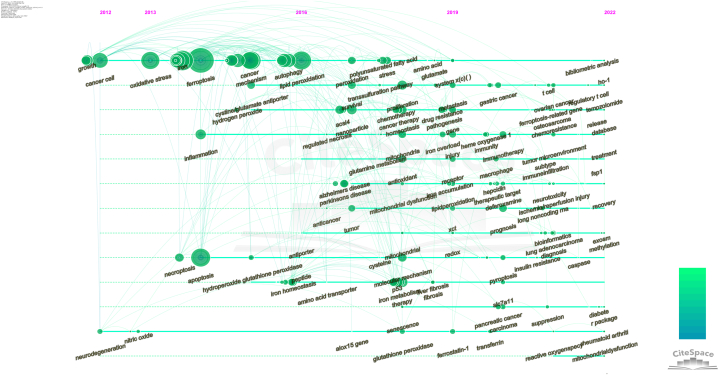
CiteSpace map of the timeline related to ferroptosis publications.

To better understand the development and features of certain research stages for a topic, the interaction among keywords and changes to the keywords listed were explored to create a timeline. Fig. 7 shows the evolution of the ferroptosis keywords based on a timeline analysis using CiteSpace software. Between 2012 and 2018, research focused on the molecular mechanisms of ferroptosis, and the main keywords were oxidative stress, iron metabolism, lipid peroxidation, molecular mechanism, cystine/glutamate antiporter, and glutamine metabolism. In the past five years, in addition to research on molecular mechanisms, the application of ferroptosis in various systemic diseases as well as cancer therapy received considerable attention, and the main keywords were prognosis, treatment, therapeutic target, recovery, gastric cancer, osteosarcoma, lung cancer, and rheumatoid arthritis. It is also noteworthy that the field of ferroptosis has also been increasingly associated with bioinformatics-related keywords in the past two years, such as bioinformatics, database, R package, and bibliometric analysis, suggesting that research associated with big data is a future direction in the field of ferroptosis that deserves the attention of academics.

### 3.6 Favoured journals

**Fig. 8:**
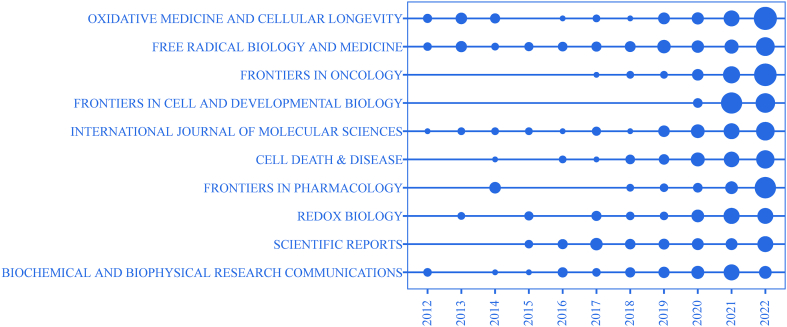
The top 10 prolific journal publications shown in temporal order.

The 10 top journals ranked by the numbers of publications and citations are presented in Table 5. *Oxidative Medicine and Cellular Longevity* with a total of 159 publications was the most prolific journal, followed by *Free Radical Biology and Medicine* with a total of 134 publications and *Frontiers In Oncology* with a total of 129 publications. The 2022 Journal Citation Reports demonstrated that *Free Radical Biology and Medicine, Frontiers In Cell and Developmental Biology, Cell Death & Disease, Frontiers In Pharmacology,* and *Redox Biology* were among the Q1 quadrant of journals. Among the top 10 most cited journals, *Cell* with a total of 11840 citations was first place, followed by *Free Radical Biology and Medicine* with a total of 7039 citations and *Nature* with a total of 6605 citations. Notably, the top 10 most cited journals were all located in Q1, except for *Oxidative Medicine and Cellular Longevity* and *Biochemical and Biophysical Research Communications*, which demonstrates that ferroptosis-related research is preferentially published in high-quality journals. The annual publication output of the top 10 most prolific journals is shown in Fig. 8. Most journals published articles on ferroptosis, contributing to an upwards annual trend, which demonstrates that ferroptosis has received considerable attention in the 10 years since it was proposed in 2012 and will be a hotspot of future research as well.

**Table 5:** Top 10 journals distributed by publications and citations.

Rank	Journal	Publications	Percentage (%)	IF(JCR2022)	JIF quartile	Journal	Total citations	Publications	Average Citations (AC)	H-index	IF(JCR2022)	JIF quartile	AC rank
1	Oxidative Medicine and Cellular Longevity	159	2.04	7.31	Q2	Cell	11840	9	1315.56	9	64.5	Q1	1
2	Free Radical Biology and Medicine	134	1.72	8.101	Q1	Free Radical Biology And Medicine	7039	134	52.53	41	7.4	Q1	8
3	Frontiers In Oncology	129	1.65	5.738	Q2	Nature	6605	11	600.45	11	64.8	Q1	2
4	Frontiers In Cell and Developmental Biology	124	1.59	6.081	Q1	Oxidative Medicine And Cellular Longevity	6316	159	39.72	30	7.31	Q2	10
5	International Journal of Molecular Sciences	116	1.48	2.598	Q2	Cell Death & Disease	5647	106	53.27	39	9	Q1	7
6	Cell Death & Disease	106	1.36	9.696	Q1	Redox Biology	5314	91	58.40	39	11.4	Q1	6
7	Frontiers In Pharmacology	101	1.29	5.988	Q1	Cell Death And Differentiation	4941	38	130.03	23	12.4	Q1	5
8	Redox Biology	91	1.16	10.787	Q1	Biochemical And Biophysical Research Communications	4522	87	51.98	30	3.1	Q2	9
9	Scientific Reports	88	1.13	4.6	Q2	Nature Chemical Biology	4356	10	435.6	10	14.8	Q1	3
10	Biochemical And Biophysical Research Communications	87	1.11	3.1	Q2	Proceedings Of The National Academy Of Sciences Of The United States Of America	4032	28	144	20	11.1	Q1	4

### 3.7 Most productive institutions

**Fig. 9:**
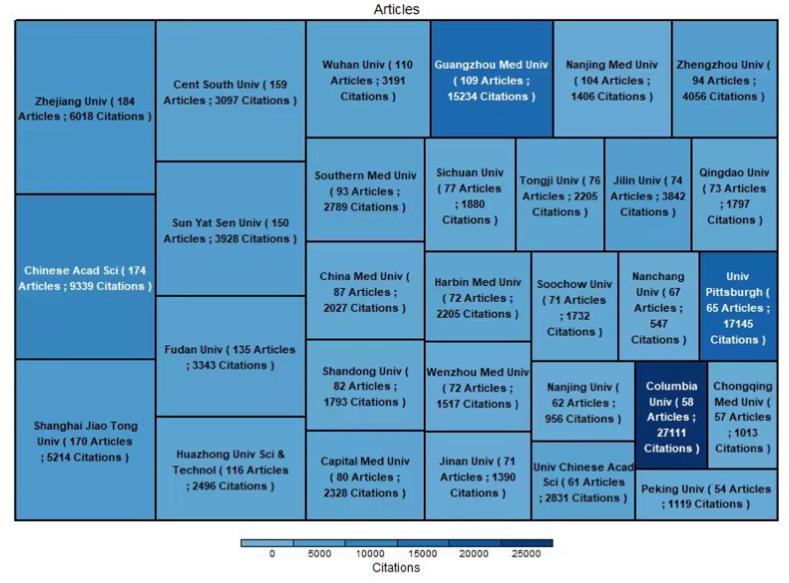
The top 10 most productive institutions.

**Fig. 10:**
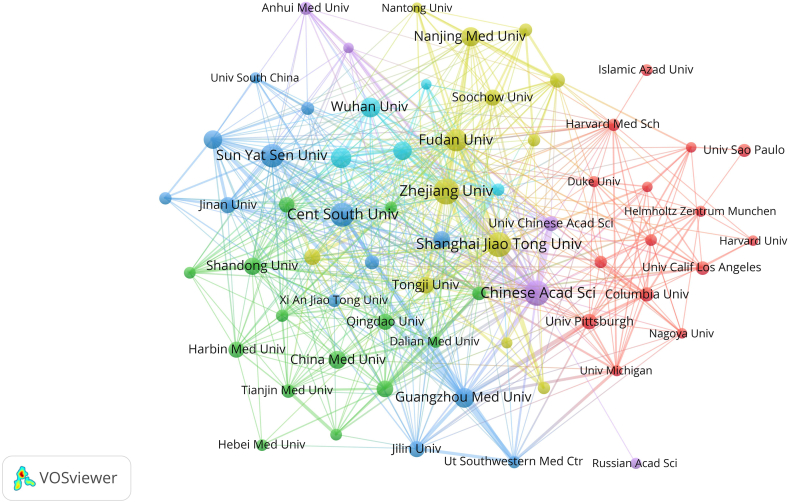
Network of institutional collaboration.

**Fig. 11:**
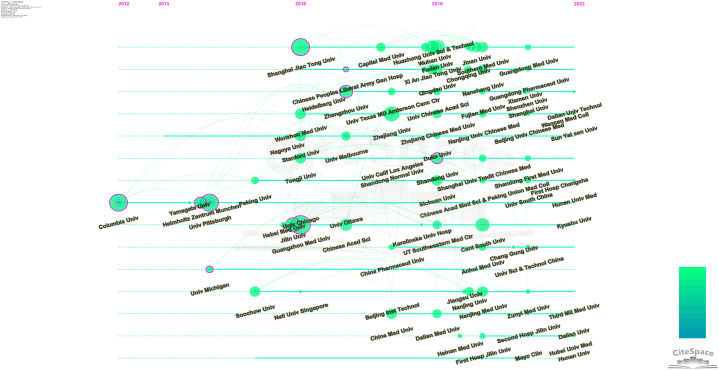
Evolution of institution publication contributions to the literature.

It can be concluded from Fig. 9 that the 30 most productive institutions were mostly in China, except for Columbia Univ (58 articles; 27111 citations) and Univ Pittsburgh (65 articles; 17145 citations), which are in the USA; these two are credited with the most total citations. The top 10 most productive institutions as determined by the number of publications were Zhejiang Univ (184 articles; 6018 citations), Chinese Acad Sci (174 articles; 9339 citations), Shanghai Jiao Tong Univ (170 articles; 5214 citations), Cent South Univ (159 articles; 3097 citations), Sun Yat Sen Univ (150 articles; 3928 citations), Fudan Univ (135 articles; 3343 citations), Huazhong Univ Sci & Technol (116 articles; 2496 citations), Wuhan Univ (110 articles; 3191 citations), Guangzhou Med Univ (109 articles; 15234 citations), and Nanjing Med Univ (104 articles; 1406 citations). The exceptional performance of Columbia Univ and Univ Pittsburgh in total citations overcame the drawback of having fewer papers published than other institutions. As a result, Columbia Univ and Univ Pittsburgh dominated the ferroptosis research field and can be considered the most authoritative institutions in this field. Institutions with more than 5 documents are shown in Fig. 10. Close interactions and collaborations were established among scholars at various institutions. Columbia Univ published the first article on ferroptosis in 2012 and has become a pioneer in the field. It was followed by Yamagata Univ, Helmholtz Zentrum Munchen and Univ Pittsburgh (Fig. 11).

### 3.8 Analysis of frequently cited articles

The top 10 most cited articles in the field of ferroptosis are listed in Table 6. With a total of 6308 citations, the article entitled “Ferroptosis: An Iron-Dependent Form of Nonapoptotic Cell Death” [1] written by Dixon, SJ, in 2012 was the most cited in the ferroptosis research field. This article, published in the leading journal *Cell*, first introduced the concept of ferroptosis, described as a novel iron-dependent mode of programmed cell death, and laid the foundation for subsequent relevant research, making its publication a meaningful milestone. The article that was cited the most frequently after that by Dixon was “Ferroptosis: A Regulated Cell Death Nexus Linking Metabolism, Redox Biology, and Disease” [17] written by Stockwell, BR in 2017, and it was also published in *Cell*. This article, which systematically illustrates the mechanisms of ferroptosis and its relationship to human diseases, is considered a programmatic guide to the study of ferroptosis. Number 3 on the list was “Lipid Peroxidation: Production, Metabolism, and Signaling Mechanisms of Malondialdehyde and 4-Hydroxy-2-Nonenal” [18] with a total of 2895 citations. It focused on two major lipid peroxidation products of omega-6 fatty acids, malondialdehyde (MDA) and 4-hydroxy-2-nonenal (4-HNE), and elaborated the metabolism, signalling mechanisms, and related pathological processes mediated via MDA and 4-HNE. The article “Ferroptosis: Process and Function” [19] was published in *Cell Death Differ*. As a classic review, it highlighted the link between the regulatory mechanisms underlying ferroptosis and physiological and pathological processes. Number 5 on the list was “Inactivation of the Ferroptosis Regulator Gpx4 Triggers Acute Renal Failure in Mice” [20] with a total of 1634 citations. This work was the first to utilize inducible Gpx4 (−/−) mice for use in relevant experiments, providing the most direct evidence at the genetic level that Gpx4 knockdown leads to cell death with the characteristics of ferroptosis and further elucidating the important role of the glutathione/Gpx4 axis in preventing lipid peroxidation-driven acute renal failure. Furthermore, the article “Ferroptosis as a p53-mediated Activity during Tumour Suppression” [21] was written by Jiang, L, in 2015 and was published in *Nature*. This work is of great importance because it reveals that the SLC7A11 gene is a target of P53 and that P53 regulates cystine metabolism, modulates the ROS response and inhibits tumour growth via ferroptosis, which further expanded the understanding of the classical antitumour factor p53. In addition, the other four highly cited articles were cited fewer than 1500 times.

**Table 6:** Top 10 cited literatures.

Rank	Authors	Citations	Article title	Journal abbreviation	Date	Volume	DOI	Pubmed ID
1	Dixon, SJ	6308	Ferroptosis: An Iron-Dependent Form of Nonapoptotic Cell Death	Cell	2012	149	10.1016/j.cell.2012.03.042	22632970
2	Stockwell, BR	3037	Ferroptosis: A Regulated Cell Death Nexus Linking Metabolism, Redox Biology, and Disease	Cell	2017	171	10.1016/j.cell.2017.09.021	28985560
3	Ayala, A	2895	Lipid Peroxidation: Production, Metabolism, and Signaling Mechanisms of Malondialdehyde and 4-Hydroxy-2-Nonenal	Oxidative Med. Cell. Longev.	2014	2014	10.1155/2014/360438	24999379
4	Xie, Y	1720	Ferroptosis: process and function	Cell Death Differ.	2016	23	10.1038/cdd.2015.158	26794443
5	Angeli, JPF	1634	Inactivation of the ferroptosis regulator Gpx4 triggers acute renal failure in mice	Nat. Cell Biol.	2014	16	10.1038/ncb3064	25402683
6	Jiang, L	1513	Ferroptosis as a p53-mediated activity during tumour suppression	Nature	2015	520	10.1038/nature14344	25799988
7	Doll, S	1459	ACSL4 dictates ferroptosis sensitivity by shaping cellular lipid composition	Nat. Chem. Biol.	2017	13	10.1038/NCHEMBIO.2239	27842070
8	Yang, WS	1368	Ferroptosis: Death by Lipid Peroxidation	Trends Cell Biol.	2016	26	10.1016/j.tcb.2015.10.014	26653790
9	Kagan, VE	1140	Oxidized arachidonic and adrenic PEs navigate cells to ferroptosis	Nat. Chem. Biol.	2017	13	10.1038/NCHEMBIO.2238	27842066
10	Bersuker, K	1129	The CoQ oxidoreductase FSP1 acts parallel to GPX4 to inhibit ferroptosis	Nature	2019	575	10.1038/s41586-019-1705-2	31634900

## 4 Discussion

The quantity and pattern of publications each year can provide insight into the progression and rate of field development. As shown in Fig. 3, since the first article on ferroptosis was published in 2012, the number of scientific publications dedicated to ferroptosis published annually has been increasing each year. In particular, the number of publications related to ferroptosis has exploded in the past 5 years, and the number of published papers exceeded 1000 in 2021. Ferroptosis has been a prominent study topic in recent years, with a promising future. However, the average number of citations from 2012 to 2022 indicates an overall declining trend, which may be because the current articles often have a smaller chance to be cited compared to previous articles.

Eleven of the 15 most cited authors were from the USA, and two were from Germany, indicating that the most authoritative scholars in the field of ferroptosis were concentrated in these two countries. Both an author's publication and citation ranking were incorporated in the criteria to identify the best authors in this field with reduced bias. After the analysis, the most accomplished academics in the field of ferroptosis, as determined by their simultaneous placement in the top 15 of the publications and citations lists, were identified as Tang, Daolin; Kang, Rui; Conrad, Marcus; Stockwell, Brent R.; and Wang, Fudi et al. In addition, the dominance factor associated with these authors was relatively low, which is because they usually are the corresponding authors. In Fig. 4, authors with a minimum of 10 publications were divided into several clusters, each representing an academic group. Considering these analyses, we suggest that particular attention be given to the academic groups represented by Tang, Daolin; Stockwell, Brent R.; Wang, Fudi, Conrad; and Marcus in the future.

In recent years, Tang Daolin, Kang Rui, and Kroemer Guido et al. have focused on the application of ferroptosis to the treatment of hepatocellular carcinoma [22], liver cancer, pancreatic cancer [23,24], and gastrointestinal tumours [25]. Furthermore, they delved into autophagy-dependent ferroptosis [26], providing insights into how selective autophagy promotes ferroptosis [[27], [28], [29]]. In 2012, Stockwell, Brent R. et al. introduced the concept of ferroptosis for the first time [1] and discovered a novel iron-dependent mode of programmed cell death. In the subsequent 10 years, Stockwell, Brent R. et al. focused on the mechanism of ferroptosis from the perspective of metabolism [30], reactive oxygen species biology [31,32], iron biology [33] and other nonclassical pathways [34,35] and systematically elucidated the key regulators of ferroptosis. Over the years, Wang Fudi et al. have made significant contributions to the diagnosis and treatment of ferroptosis in neurodegenerative diseases such as traumatic brain injury [36] and Alzheimer's disease [37]; digestive diseases such as hepatic ischaemia‒reperfusion injury [38] and fatty liver [39,40]; cardiovascular diseases such as cardiomyopathy [41,42] and heart failure [43]; and skeletal muscle diseases of the locomotor system [44]. Furthermore, utilizing cutting-edge technologies and platforms in life sciences, medicine and public health, Team Wang Fudi has made breakthroughs in elucidating the molecular mechanisms underlying the steady-state metabolism of trace elements [45] such as iron, zinc [46,47], manganese and copper [48] and in the prevention and control of major chronic diseases. In addition, Conrad, Marcus et al. focused on the role of GPX4 [49], FSP1 [50,51], and vitamin K [52] in the regulation of ferroptosis and demonstrated the important role of these key factors with models of animal diseases [53].

China was the country with the highest publications and citations, followed by the USA. The scientific output by authors in China and the USA together accounted for 58% of all publications, which indicates that China and the USA were the leading countries in the field of ferroptosis research. In addition, the average number of citations from Germany and the USA was much higher than that of other countries, which demonstrates that Germany and the USA had high-quality research achievements, and thus have received a great deal of attention. In contrast, the average number of citations of papers from China lagged behind that of other countries, perhaps because most of the publications from China were published in the past 5 years. These data also suggests that Chinese scholars should pay more attention to the value and quality of their published articles. Overall, China, the USA, and Germany have been the front-runners in the field of ferroptosis.

*Oxidative Medicine and Cellular Longevity* was the most prolific journal with a total of 159 publications, but *Oxidative Medicine and Cellular Longevity* publications were in Q2 of the 2022 JCR. Since ferroptosis was proposed in 2012, *Free Radical Biology and Medicine* has published a stably increasing number of papers each year, with a total of 134 articles, becoming the second most prolific journal. In addition, *Free Radical Biology and Medicine* has a journal impact factor of 8.101, which puts in Q1 in the 2022 JCR. Overall, *Free Radical Biology and Medicine* is a prestigious journal in the field of ferroptosis, with bright prospects for the future. Fig. 8 shows that the top 10 journals with the most publications showed an increasing trend in the scientific output as measured by the number of ferroptosis-related articles published every year. In terms of citations, *Cell* and *Nature* were the journals with the highest number of average citations, and the number of citations was much higher for these two journals than for other journals, indicating that they have been the most authoritative journals in the field of ferroptosis research. As indicated by the 10 most cited journals shown in Table 5, six journals were cited 5000 times, and except for *Oxidative Medicine And Cellular Longevity* and *Biochemical And Biophysical Research Communications*, all the journals were in Q1 of the 2022 JCR. Moreover, the 10 most cited articles were mostly from high-impact journals, demonstrating that ferroptosis-related research is highly recognized in the academic community worldwide.

Fig. 9 shows the 30 most prolific institutions. The size of the rectangle is proportional to the number of publications, and the intensity of the colour is proportional to the number of citations. There is relatively minor variation in the scientific output among different institutions but a significant difference in the number of citations among different institutions. Twenty-eight of the top 30 most prolific institutions were in China, but the number of citations for Chinese papers was relatively low, while that of two institutions (Univ Pittsburgh and Columbia Univ, ranked 25 and 28 in the number of publications) in the USA was the highest. In addition, Columbia Univ, as the publisher of the earliest and most cited article on ferroptosis, has become the pioneer in this field. Columbia Univ and Univ Pittsburgh, owing to their exceptionally high number of citations at 27,111 and 17,145, respectively, can be considered the most authoritative institutions in the field of ferroptosis despite the relatively low number of publications attributed to them. As shown in Fig. 10, most of the research institutions with a minimum of 5 publications were in China, and institutions have relatively close cooperation with each other, which may be the reason for the surge of Chinese publications in recent years. Most of the institutions in the USA were clustered in the red group, while most of the Chinese institutions were distributed in clusters with other colours, suggesting that scholars in institutions in China and the USA tend to cooperate more frequently with others at institutions in their own countries and exhibit relatively low cross-country cooperation. As shown in Fig. 11, the ranking of institutions in China was largely increased in the past 5 years, which may explain the relatively low number of citations; however, it also suggests that Chinese institutions may show greater potential in the future.

## 5 Hotspots and frontiers

Keywords describe the main topics of an article and can help us quickly grasp the research direction of an article. Table 4 shows that the keywords used with the highest frequency were lipid peroxidation, oxidative stress, iron, iron metabolism, inflammation, reactive oxygen species, cancer, and prognosis. Based on this keyword co-occurrence analysis, the topics that are research hotspots and the evolution of research hotspots within a certain field can be ascertained. A cluster analysis was performed on the keywords, and ultimately, four clusters were produced, and they are presented in different colours. Then, we identified research hotspots and frontiers in the field of ferroptosis based on a timeline analysis. The key results are presented in this section.

### 5.1 The metabolic pathways underlying the ferroptosis

#### 5.1.1 The amino acid metabolism pathway

System Xc^−^ (cystine/glutamate antiporter) is a reverse transporter protein widely distributed on the cell surface and is a heterodimer consisting of a heavy chain (SLC3A2) and a light chain (SLC7A11). It mediates a 1:1 exchange between intracellular glutamate and extracellular cystine to maintain intracellular redox homeostasis by introducing exogenous cystine into cells. The cystine that enters cells is reduced to cysteine (l-cysteine), which is needed for glutathione (GSH) synthesis [54,55]. GSH is a tripeptide and the most powerful intracellular antioxidant that plays an important role in the reduction of many peroxides [56]. A GSH deficit makes cells susceptible to oxidative stress. Glutathione peroxidase 4 (GPX4) function as a GSH-dependent lipid peroxidase to prevent ferroptosis by reducing the peroxide bond of peroxides to hydroxyl groups, thereby converting peroxides to nontoxic lipid alcohols [4]. As the activity of system Xc^−^ is inhibited, cells cannot take up a sufficient amount of cysteine, resulting in insufficient GSH synthesis, decreased GPX4 activity, and accumulation of H_2_O_2_ generated via intracellular metabolism and triggers Fenton reaction with Fe^2+^ to produce an excess of oxygen radicals, ultimately leading to ferroptosis.

#### 5.1.2 The lipid metabolism pathway

The principal feature of ferroptosis is the accumulation of iron-dependent lipid peroxides. The substrates for the lipid peroxidation reaction are fatty acids, which include polyunsaturated fatty acids (PUFAs) and monounsaturated fatty acids (MUFAs). PUFAs are more prone to peroxidation due to the extremely weak C–H bond between adjacent C=C units in PUFAs, which can be cleaved by a powerful oxidant to generate a radical [2,16]. Notably, free PUFAs are not drivers of ferroptosis, and only activated PUFAs bound to membrane phospholipids (PLs) exert lethal lipid peroxidation effects [16]. Thus, the accumulation of PUFA-PLs is a marker of ferroptosis, and the amount of intracellular PUFA-PLs determines the susceptibility of cells to ferroptosis [57,58]. In addition, not all PUFAs are susceptible to peroxidation. In 2017, Valerian E. Kagan et al. reported that specific phospholipids, namely phosphatidylethanolamines (PEs) with arachidonoyl (AA, 20:4) or adrenoyl (AdA, 22:4) PUFA tails, are more likely to induce ferroptosis than other PUFAs [6,35]. The enzymatic reaction of lipid peroxidation involves three key enzymes: acyl-CoA synthetase long-chain family member 4 (ACSL4), lysophosphatidylcholine acyltransferase 3 (LPCAT3), and lipoxygenases (LOXs). ACSL4 catalyses the binding of free AA or AdA to coenzyme A (CoA) in a substrate-specific manner to form the derivative fatty acyl-CoA. LPCAT3 then esterifies Fatty acyl-CoA to the sn2 position of a lysophospholipid (lysoPL), generating a phospholipid (PL) [2]. The LPCAT family members exhibit distinct substrate preferences. Michael Kazachkov et al. identified that LPCAT3 prefers to esterify AA-CoA to lysoPL and therefore plays a significant role in ferroptosis [59]. However, there has been no comprehensive research on the function of other LPCAT family members in ferroptosis [2]. Subsequently, LOXs can catalyse the peroxidation of membrane phospholipids and accelerate ferroptosis. In a P53-induced ferroptosis model, the P53 protein activated arachidonate-12-lipoxygenase (ALOX12), thereby triggering ferroptosis, and the process was not dependent on GPX4, providing a new method of preventing ferroptosis [60].

#### 5.1.3 The iron metabolism pathway

Iron is one of the essential trace elements in the human body, and iron deficiency or overload can lead to cellular and even organismal damage. Iron is mostly derived from food but may also be retrieved from the liver and aged red blood cells (RBCs) [61]. Dietary iron, mainly trivalent iron ions (Fe^3+^), is absorbed by the intestinal epithelial cells of the duodenum and upper jejunum and bound to transferrin (TF) to form a TF-Fe^3+^ complex, which is circulated through the bloodstream and thus transported to various tissues and organs. Fe^3+^ enters a cell via the action of transferrin receptor-1 (TFR-1) on the cell membrane, and intracellular STEAP3 reduces Fe^3+^ to Fe^2+^, which is followed by a series of biochemical reactions. Ferritin is the major iron-storage protein and consists of ferritin heavy chain 1 (FTH1) and ferritin light chain (FTL). In addition, excess Fe^2+^ is released into the labile iron pool (LIP) in the cytoplasm via the action of divalent metal transporter 1 (DMT1).

Iron overload is an important contributor to ferroptosis. Excessive Fe^2+^ contributes to intracellular oxidation reactions and Fenton reactions, generating large amounts of free radicals and reactive oxygen species (ROS), which in turn peroxidize PUFAs on the cell membrane and generate toxic lipid peroxides, leading to the destruction of the cell membrane structure and eventually resulting in ferroptosis [61,62]. In addition, Fe^2+^, as a cofactor of several enzymes involved in lipid peroxidation reactions (such as LOXs and P450 oxidoreductase), can enhance the activity of these enzymes and promote ferroptosis [2,63]. Correspondingly, the regulatory mechanisms of intracellular iron metabolism have been relatively well established. Cellular iron homeostasis is primarily controlled by the regulatory proteins IRP1 and IRP2, which control the posttranscriptional regulatory mechanisms involved in intracellular iron uptake, storage and release [64]. Moreover, the intracellular iron content determines the sensitivity of cells to ferroptosis. OTUD1 prevents ferroptosis by deubiquitinating and stabilizing IREB2. This results in an increase in TFRC-1 expression and a decrease in iron uptake [65].

#### 5.1.4 Other metabolic pathways

Ferroptosis may be regulated by other pathways in addition to those described above. Nuclear factor E2-related factor 2 (Nrf2) is a key regulator of the intracellular antioxidant response, and it regulates the expression of almost all genes associated with ferroptosis, including genes involved in the intracellular regulation of antioxidants, iron and various intermediate metabolites [66]. Hui Dong et al. demonstrated that Nrf2 attenuated ferroptosis injury by regulating the expression of SLC7A11 and HO-1 in an acute lung injury model [67]. Activation of the Nrf2-Keap1 signalling pathway upregulated system Xc^−^ to promote cell proliferation and transformation [68]. In addition, it has been reported that NAD(P)H/FSP1/CoQ10 and GCH1/BH4/DHFR are GPX4-independent antioxidant signalling pathways. Ferroptosis suppressor protein 1 (FSP1) uses NADPH to reduce ubiquinone (CoQ10) to ubiquinol (CoQH2), which is an antioxidant that traps lipophilic free radicals, prevents the accumulation of lipid peroxides, and inhibits ferroptosis [5]. GTP cyclohydrolase 1 (GCH1) is the rate-limiting enzyme for tetrahydrobiopterin (BH4) synthesis. Dihydrofolate reductase (DHFR) is a key enzyme in the dihydrobiopterin (BH2) synthesis pathway that yields BH4. BH4 functions as a lipophilic antioxidant, similar to CoQ10, and inhibits lipid peroxidation [69]. Furthermore, GCH1 can also increase the abundance of reduced CoQ10 in cell membrane lipids, exhaust PUFA-PLs and suppress ferroptosis [16].

### 5.2 Role of ferroptosis in diseases

Through the timeline analysis, we can see that during 2012–2018, research on ferroptosis was focused mainly on molecular mechanisms. With the deepening of relevant basic research, the research direction of ferroptosis has gradually shifted from basic research to research on clinical treatments in the past 5 years. Studies have reported an important role for ferroptosis in cancer and neurological, cardiovascular system and ischaemia‒reperfusion diseases.

#### 5.2.1 Ferroptosis inducers trigger the death of cancer cells

Since the inception of the field, ferroptosis has been connected to cancer. Because metabolism in cancer cells often have more active and produce a larger ROS population, it is possible that cancer cells may have a greater propensity to undergo ferroptosis [70]. It has been demonstrated that cancer cells often rely on high iron concentrations, which might make them even more susceptible to ferroptosis [71].

Currently, chemotherapy and radiotherapy are the major treatments for malignant tumours; however, resistance to chemotherapy and radiotherapy often leads to treatment failure. Ferroptosis inducers are effective in killing multiple tumour cells, and in combination with traditional chemotherapy drugs, they can enhance the efficacy of treatment and slow the acquisition of drug resistance by tumour cells. Tanshinone IIA (Tan IIA) upregulated the expression of the ferroptosis markers Ptgs2 and Chac1 in gastric cancer cells and downregulated the expression of SCL7A11 by mediating the expression of P53, which in turn caused a decrease in intracellular GSH and cysteine levels and an increase in ROS levels, inhibiting the proliferation of gastric cancer cells [72]. The anticancer drug sorafenib triggers endoplasmic reticulum stress and ferroptosis by inhibiting system Xc^−^ activity [73]. Erastin/sorafenib synergize with cisplatin to enhance the suppressive effect of the cisplatin-mediated Nrf2/SLC7A11 pathway, alter drug resistance in non-small cell lung cancer, and effectively trigger ferroptosis [74]. Radiotherapy not only causes DNA damage but also increases intracellular ROS levels, which indicates that it can activate ferroptosis and increase the efficacy of radiotherapy. The ferroptosis inducers IKE and RSL3 synergistically interact with radiation therapy, enhancing the effects of radiation on the cytoplasm to increase its effectiveness [75]. Immunotherapy is a tumour treatment approach that has been applied in recent years. It activates and strengthens the body's intrinsic immune system to kill tumour cells or inhibit their proliferation. However, immune escape is a huge challenge to effective tumour treatment. CD8^+^ T cells not only exert immunotherapeutic effects through the perforin-granzyme and Fas-Fas ligand pathways but also release interferon-gamma (IFNγ), which inhibits the expression of the two subunits, SLC7A11 and SLC3A2, of system Xc^−^. This prevents the absorption of intracellular cystine and the synthesis of GSH, increasing the ferroptosis rate of cancer cells [76].

#### 5.2.2 Ferroptosis inhibitors prevent cell death in normal body cells

Ferroptosis inhibitors can prohibit normal cell death in the body and prevent damage to vital organs during pathophysiological processes, which may become a future therapeutic measure for various systemic diseases. Atherosclerosis (AS) is the basic underlying condition of many cardiovascular diseases and is characterized by lipid metabolism disorders. Tao Bai et al. found that the ferroptosis inhibitor ferrostatin-1 may alleviate AS by attenuating lipid peroxidation and endothelial dysfunction [30]. Endothelial progenitor cells secreting extracellular vesicles (EPC-EVs) transfected with miR-199a-3p inhibited specificity protein 1 (SP1), thereby suppressing endothelial cell ferroptosis and delaying AS progression. Heart failure (HF) is characterized by cardiomyocyte hypertrophy and fibrosis. Cardiomyocytes are highly susceptible to free iron overload, which disrupts cardiomyocyte homeostasis and triggers heart failure. In cardiomyocytes with ferritin heavy chain (FTH) deficiency, the content of cellular unstable iron is increased, promoting increases to intracellular ROS levels and exacerbating lipid peroxidation and ventricular remodelling. This phenotype was rescued after the application of ferrostatin-1, which confirmed the role of ferroptosis in the pathological process underlying HF [77]. Chen et al. found that knockdown of TLR4 and NADPH oxidase 4 (NOX4) in rats with heart failure resulted in the elevated expression of GPX4 and FTH-1, reduced the free iron level in cardiomyocytes, significantly attenuated left ventricular remodelling, and prevented cardiomyocyte death. Based on bioinformatics analysis, TLR4 is a molecule upstream of NOX4, confirming that the TLR4-NOX4 pathway plays an important role in the pathogenesis of heart failure [78]. Parkinson's disease (PD) is a complicated disorder characterized by progressive dopaminergic neuronal degeneration. Bruce Do Van et al. showed that erastin induces ferroptosis in neuronal precursor cells, but iron chelators, such as ferrostatin-1 derivatives, inhibited dopaminergic neuronal damage [79]. Ferroptosis is closely associated with hepatic ischaemia‒reperfusion (I/R) injury. Fang et al. identified a previously unknown anti-ferroptosis gene, malic enzyme 1 (Me1), in rats with hepatic ischaemia‒reperfusion injury and demonstrated that downregulation of Me1 led to the depletion of NADPH in hepatocytes, which in turn led to cysteine deficiency and triggered GSH synthesis, increasing the rat cell susceptibility to ferroptosis. Subsequently, hepatic I/R injury was alleviated by treating the rats with the substrate of Me 1. These findings indicate that l-malate shows promise as a clinical therapy to protect the liver against I/R injury [80].

In addition, the effects of iron chelators and lipophilic RTA on various systemic diseases have been proven in animal models. However, there is still a significant risk associated with the use of iron chelators in a systemic manner [81]. This potential problem needs to be solved in the near future by scientists in the field of ferroptosis.

### 5.3 Interdisciplinary ferroptosis and big-data research may become a new frontier

Notably, bioinformatics-related keywords were identified in articles published in the past two years (Fig. 7); these keywords include bioinformatics, database, R package, and bibliometric analysis, which also indicates that ferroptosis and big-data as an interdisciplinary field will be a new frontier.

## 6 Limitations

This bibliometric study has some limitations. First, the documents studied were limited to those in the Web of Science core collection database and thus only included some of the articles produced in this field worldwide, which may have resulted in biased data. Second, we might have ignored certain articles on ferroptosis if our research keyword was not in the title of a publication, resulting in an inaccurate analysis.

## 7 Conclusion

Since the first article on ferroptosis was published in 2012, the number of scientific publications produced annually has been increasing yearly. The academic communities represented by Tang, Daolin; Stockwell, Brent R.; Wang, Fudi; and Conrad, Marcus were the most authoritative. China, USA, and Germany were the front-runners in the field of ferroptosis. *Free Radical Biology and Medicine* was the main publication related to ferroptosis research, and *Cell* and *Nature* were the most influential journals. Columbia Univ and Univ Pittsburgh were the institutions that received the most attention. Currently, research on ferroptosis is focused on molecular mechanisms and their effect on various diseases, which will be a hotspot for future research. In addition, interdisciplinary ferroptosis and big-data research is expected to become a new frontier.

## Declarations

### Author contribution statement

Bingzhou Ji: Conceived and designed the experiments; Wrote the paper. Guang Yang: Hongfu Jin: Performed the experiments. Xu Liu: Hengzhen Li: Analyzed and interpreted the data. Linyuan Pan: Wenhao Lu: Heyuan Zhu: Contributed reagents, materials, analysis tools or data. Yusheng Li: Conceived and designed the experiments.

### Data availability statement

Data will be made available on request.

## Declaration of competing interest

The authors declare no conflict of interest.
